# Between Openness and Privacy in Genomics

**DOI:** 10.1371/journal.pmed.1001937

**Published:** 2016-01-12

**Authors:** Effy Vayena, Urs Gasser

**Affiliations:** 1 Health Ethics and Policy Lab, Institute of Epidemiology, Biostatistics and Prevention, University of Zurich, Zurich, Switzerland; 2 Berkman Center for Internet & Society, Harvard Law School, Harvard University, Cambridge, Massachusetts, United States of America

## Abstract

With the prospect of genomic data becoming ever more easily available, Effy Vayena and Urs Gasser discuss how we could balance making the most of its benefits with reducing its risks to privacy.

**Summary Points**

- Advancing genomic research depends on the accessing and sharing of genomic data. However, the increasing need for sharing escalates the tension between genomic privacy and openness.
- Promoting openness while protecting privacy is a challenge that cannot be overcome only with technical solutions such as encryption and differential privacy. Although such solutions are crucial, we still need to confront some fundamental normative tensions that are intensified in the era of genomics and big data. Here are at least three:
- The right to genomic privacy is not an absolute right. If privacy is understood as control over information or data, privacy is not about maximal levels of control, but rather about reasonable measures of control.
- Although individual control is necessary, it is not a sufficient safeguard of privacy. Individuals’ willingness to be open about their data does not obviate responsibility for reducing privacy risks on the part of the data users.
- Data governance models, such as data cooperatives, that enable meaningful and continuous roles of the individuals whose data are at stake hold promise for reconciling privacy and openness.

Quickly sequencing your DNA on your mobile phone is science fiction but might not be for long—companies are already investing in the idea [1,2]. Genomic data are being generated at ever higher speeds: clinics, personal genomic companies like 23andme.com and ancestry.com, pharmaceutical industry labs, funding agencies, and public–private initiatives are all grappling with the implications. Genomics, along with proteomics, metabolomics, and other omics, promises, as President Obama recently put it, to give “us all access to the personalized information we need to keep ourselves and our families healthier” [3].

Generating genomic data is nothing without translating it into practical heath care measures [4]. Breakthroughs depend on linking genomic data with other omics and phenotypic data [5,6]. Yet, genomic datasets have become subject to access restrictions on technological, cultural, ethical, and legal grounds. Restrictive policies diminish the data’s utility by creating bottlenecks, hampering discoverability, imposing burdensome access authorization processes, and delaying research [7]. Understandably, the scientific community pleads for openness, both from individuals supplying their genomes and from other researchers holding datasets. Openness here encompasses everything from conditional access for limited groups to data sharing in publicly available repositories or even open online platforms.

Several important initiatives promoting this openness have emerged. The Personal Genome Project has long been at the forefront [8], while the National Institutes of Health’s (NIH’s) recent policy promotes the sharing of large-scale genomic datasets [9], and the Global Alliance for Genomics and Health is putting in place foundations to share this data globally [10]. Patient- and citizen-led initiatives are calling for access to personal genomic and health data, allowing individuals to decide on additional uses (e.g., freethedata). Online platforms such as openhumans.org and openSNP.org allow people to make their genomic data publicly accessible. These developments indicate growing pressure to open up genomics in order to achieve the objectives that led to the generation of the data in the first place.

If genomic openness is such an imperative, why does genomic data access and sharing remain heavily contested and underdeveloped? The answer to this question is complex, but one vexing challenge is often singled out as the biggest inhibiter of genomic openness: openness is widely regarded as having potentially adverse implications for privacy, and it cannot be denied that we are still grappling with some fundamental questions about the symbiosis of privacy and openness. Would more openness inevitably lead to discrimination and various forms of social exclusion? Is genomic openness a real threat to privacy? Even if legal protections against discrimination in employment and health insurance are in place, does this exhaust the ways in which health and genomic privacy breaches can be harmful? What is the meaning of privacy in a future Internet of Things that includes an internet of omics in which data are openly accessible? Can our genomic privacy be secured if our data are the property of corporations motivated by profit? Given state surveillance in the digital era, is it prudent to expose yet another type of personal data?

Technical fixes are one strategy for overcoming the tension between openness and privacy. For example, anonymization of genomic data has been advanced as a reliable means of protecting privacy: regulators recommend this in order to justify increasing access. However, recent research has demonstrated that it is possible for anonymous data in genomic research projects to be reidentified [11]. Furthermore, identifiability itself is often desirable in the context of personalized medicine and research, so anonymity (even partial) is not always ideal. There is considerable interest in developing technologies that can ensure genomic privacy by employing encryption, differential privacy, etc. [12,13]. These laudable efforts will hopefully lead to a toolbox of privacy solutions capable of meeting many of the privacy challenges surrounding genomic data [14]. However, many of these approaches are still aiming for a technological translation of privacy norms that emerged in the pregenomic and preinternet era. We argue that the real breakthrough in reconciling privacy and openness in genomics requires confronting head-on some fundamentally normative tensions that are escalated in the era of genomics and big data. We suggest three points that will play a crucial role in navigating between privacy and openness.

## The Right to Genomic Privacy Is Not Absolute

An individual’s right to privacy is widely assumed to be antagonistic to the health-related public goods resulting from openness. In this view, an individual’s right to privacy may have to be infringed upon to promote the public good. There is a legitimate public interest in genomic data, it is believed, justifying such infringements for the greater good. But this view of the relationship between the right to privacy and the public good of health is too crude. Two observations are important here.

First, one common understanding of genomic privacy is that it is a form of information privacy. The information derived from personalized genomic analyses—treated as health information under current privacy laws—can be among the most identifiable of personal data. In this understanding, the person whose genome is in question has the right to control the flow of identifiable information as part of exercising his or her autonomy.

Legitimate research interests do not discount the right of the individual to privacy. In fact, it is the acknowledgment that individuals are entitled to control their personal information that allows such research to take place. For example, individuals may be prepared to waive privacy rights in order to secure personal and communal health benefits. Several studies have shown that people care about their privacy (strongly believing that it is their right) but that they are willing to participate in genomic research, sacrificing some privacy to do so [15,16]. People negotiate their interests in different and dynamic ways that reflect both their views about privacy and their individual circumstances. Researchers and institutions face an obligation to empower individuals to negotiate these fluid privacy boundaries based on a realistic cost and benefit analysis. This requires new approaches to transparency and accountability beyond simple information disclosure practices. For example, there is increasing attention paid to the accountability of algorithms that are used in data analyses, which is relevant to genomic analyses as well [17].

The second point is that privacy does not give individuals absolute control over their information, only reasonable measures of control [18]. The notion of reasonableness is contingent on a number of practical factors and is shaped by the presence of several moral norms such as the common good, transparency, and reciprocity. Reasonable control of data does not exclude certain degrees of openness that would permit data uses for a legitimate public interest, such as some types of genomic research. Understood in this way, privacy need not be routinely infringed upon to pursue the common good of health [19]; instead, the contours of that right can be shaped so as not to impose unreasonable burdens. This requires individuals having reasonable expectations about the flow of their information, necessitating efforts to build awareness and genomic literacy. This is a shared responsibility, including—but going beyond—the research community.

## Individual Control Is a Necessary but Insufficient Safeguard

The success of genomic initiatives is dependent on gaining public trust. In 2014, care.data, a United Kingdom initiative aimed at making aggregated national medical data available for health research, came to an abrupt standstill due to widespread public suspicion. It has been argued that the failure of the care.data initiative was the result of a prior failure to secure the social license necessary for such a project, a license that cannot be secured merely through compliance with formal regulation [20]. Although not a purely genomic project, care.data is a landmark case and a rich source of insights about the future of openness and privacy. Consider, for example, informed consent, a common method of giving individuals control of their data and also a means of establishing trust. Several consent models and approaches have been developed with varying advantages and limitations [21,22]. Care.data’s failure shows that satisfying the relevant legal requirements does not fulfill all relevant ethical demands and is not always sufficient to build trust. Consent can go as far as including explicit reference to limited, if any, privacy protections. Where does this leave privacy rights, be it the rights of the individual whose genomic data are in question or those of their relatives about whom much can be inferred from these data, even if they never agreed to sharing it [23]? How much “control” can one exercise over unknown future possibilities?

Advocates of “open consent” have argued that a pragmatic stance towards privacy could take the form of an honest and explicit communication of risks to those whose data are being used [24]. Those willing to be more open with their data would accept additional privacy risks. By being transparent and honest, though, we only establish an agreement about the existence of privacy risks, not whether taking such risks is justified or the extent to which institutions bear further responsibilities for privacy protection. As individuals come to have increasing options to open their genomic data to research in public, corporate, or public–private initiatives, the question of institutional responsibility becomes more pressing. This forces us to think of privacy beyond individual control with its focus on consent. As argued earlier, the notion of control over personal information is tightly linked to individual autonomy, but privacy is not only about autonomy, despite being intertwined with it. Privacy is a state of being, valuable and worthy of respect in and of itself [25,26]. In our view, this understanding of privacy is more comprehensive and gives rise to further obligations related to privacy protections. Individuals who choose to make compromises regarding their privacy are not thereby stripped of their right to it. (The same clearly applies to the relatives of those whose genomic data have been used.) In other words, voluntary genomic openness on the part of individuals does not obviate responsibility for reducing privacy risks on the part of the data users. This notion of continued responsibility has emerged in other areas of information privacy, governed by different legal safeguards, and is well illustrated in the words of Justice Sotomayor on a case related to personal data: “it may be necessary to reconsider the premise that an individual has no reasonable expectation of privacy in information voluntarily disclosed to third parties” [27].

Elrich et al. have recently advanced a case for further responsibilities being placed on the users of personal data. In an attempt to redefine genomic privacy, they suggest moving from a privacy focus to a trust-enabling framework grounded in transparency, increased control, and reciprocity [28]. While creating a trusted environment is undoubtedly necessary for research, this cannot be achieved without engaging with the right to privacy. Securing the right to privacy is necessary for trustworthiness. Attempts to define genomic privacy cannot be divorced from larger debates about privacy in the digital era. All personal data are subject to the vulnerabilities and strengths that come with digitalization.

## Next-Generation Data Governance Models

Crucial to negotiating openness and privacy is the governance put in place for any given genomic project. The focus on genomic governance has been discussed in the literature, with a number of interesting proposals specifically for genomic biobanks [29–32]. Most of these recognize the limitations of informed consent and the constantly changing demands and goals of genomic research. There is limited empirical evidence as to how any of these proposals might work in practice, and exploring and refining the principles of good genomic governance remains at a rudimentary stage of development. Principles of governance such as reflexivity [33], adaptivity, and deliberation [31] can serve as foundations for a plurality of models. Genomic data are collected in a variety of repositories: they may be held by a corporate or public institution, national initiatives of large scale, etc. A one-size fit all governance model is unlikely to suit all of them well. The objectives of accountability, trustworthiness, and participation remain constant but can be pursued by a variety of different schemes.

A crucial issue in the formation of any model is whether it gives a sufficient role to those whose data are at stake. This is not a U-turn back to consent but the challenge of ensuring participants are involved in the entirety of what happens with their data. That means not merely involvement in how one’s data are used but also, for example, whether access to the entire dataset for specific purposes should be granted and how benefit sharing or intellectual property is negotiated. This approach achieves a number of goals, the most relevant here being that for some degree of openness to become the default, privacy should remain contextual, with controls in place to ensure that every individual involved has a say. Returning to the point made earlier, privacy interests are negotiable, but the right to privacy is still respected.

Clearly, working out the operationalization of such models is not easy, and there is a need for creative and imaginative solutions. One new and exciting proposal is the formation of national personal data cooperatives, owned and governed by citizens and independent from governments or corporations [34]. While this proposal is not explicitly focused on genomics, its ambition is to include the whole “tapestry of [one’s] high-valued information sources” [35] that would ultimately make genomic research even more effective. It is envisaged that members of the cooperative would authorize the use of their pooled data but would retain a say (and a vote) about the general uses of their data and the direction of the cooperative as a whole. Their membership gives them an ongoing role, including sharing the benefits resulting from the use of their data. In this model as in others, practical issues need to be resolved, including the creation of easy and standardized pathways for researchers and research institutions to join such a data cooperative or forms of dividends to be returned to participants. However, several of these questions, at least from a legal perspective, are not entirely new and have been dealt with, mutatis mutandis, in the context of very large cooperatives.

## Conclusion

Given the limits of the consent approach that is at the core of conceptualizing “privacy as control,” there is a need to develop new frameworks for genomic privacy that support enough openness for genomic research. Such frameworks should be based on the understanding that privacy protections are a mutual and continuous responsibility, which in turn requires more openness. Much work still needs to be done, and silver-bullet solutions are not within reach, but recent initiatives suggest outlines for next-generation frameworks and practices. These efforts go beyond existing approaches in at least three ways. First, traditional legal and organizational safeguards that have focused largely on the collection of data (through consent requirement) become complemented by safeguards applying to all stages of the information lifecycle in research and data sharing settings. Second, legal and organizational privacy measures are supported by new and advanced computational instruments, such as differential privacy, data tag systems, or license generators helping individuals, researchers, and intermediaries to identify and manage privacy and data security risks. Third, new efforts emphasize systemic awareness raising about both privacy and openness along with their risks of harm and benefit.

Privacy and openness are rich, complex, and related norms. It is overly simplistic to think of them as static, one dimensional, or as inherently antithetical. Advances in genomics and big data have urged us to examine our normative understanding of them, how they relate to one another, and the conditions of their symbiosis. Promoting genomic research and human well-being will require keeping faith with both openness and privacy.

## Abbreviations

NIH: National Institutes of Health
